# Enhancing Recurrence-Free Survival Prediction in Hepatocellular Carcinoma: A Time-Updated Model Incorporating Tumor Burden and AFP Dynamics

**DOI:** 10.1245/s10434-025-17303-y

**Published:** 2025-04-16

**Authors:** Miho Akabane, Jun Kawashima, Abdullah Altaf, Selamawit Woldesenbet, François Cauchy, Federico Aucejo, Irinel Popescu, Minoru Kitago, Guillaume Martel, Francesca Ratti, Luca Aldrighetti, George A. Poultsides, Yuki Imaoka, Andrea Ruzzenente, Itaru Endo, Ana Gleisner, Hugo P. Marques, Sara Oliveira, Jorge Balaia, Vincent Lam, Tom Hugh, Nazim Bhimani, Feng Shen, Timothy M. Pawlik

**Affiliations:** 1 Department of Surgery, The Ohio State University Wexner Medical Center and James Comprehensive Cancer Center, Columbus, OH USA; 2 Department of Hepatobiliopancreatic Surgery, APHP, Beaujon Hospital, Clichy, France; 3 Department of General Surgery, Cleveland Clinic Foundation, Cleveland, OH USA; 4 Department of Surgery, Fundeni Clinical Institute, Bucharest, Romania; 5 Department of Surgery, Keio University, Tokyo, Japan; 6 Department of Surgery, University of Ottawa, Ottawa, ON Canada; 7 Department of Surgery, Ospedale San Raffaele, Milan, Italy; 8 Department of Surgery, Stanford University, Stanford, CA USA; 9 Department of Surgery, University of Verona, Verona, Italy; 10 Department of Gastroenterological Surgery, Yokohama City University School of Medicine, Yokohama, Japan; 11 Department of Surgery, University of Colorado, Denver, CO USA; 12 Department of Surgery, Curry Cabral Hospital, Lisbon, Portugal; 13 Department of Surgery, Westmead Hospital, Sydney, NSW Australia; 14 Department of Surgery, School of Medicine, The University of Sydney, Sydney, NSW Australia; 15 The Eastern Hepatobiliary Surgery Hospital, Second Military Medical University, Shanghai, China

**Keywords:** Hepatocellular carcinoma, Hepatectomy, Alpha-fetoprotein, Tumor burden score, Recurrence-free survival, Time-varying Cox model

## Abstract

**Background:**

Existing models to predict recurrence-free survival (RFS) after hepatectomy for hepatocellular carcinoma (HCC) rely on static preoperative factors such as alpha-fetoprotein (AFP) and tumor burden score (TBS). These models overlook dynamic postoperative AFP changes, which may reflect evolving recurrence risk. We sought to develop a dynamic, real-time model integrating time-updated AFP values with TBS for improved recurrence prediction.

**Patients and Methods:**

Patients undergoing curative-intent hepatectomy for HCC (2000–2023) were identified from an international, multi-institutional database with RFS as the primary outcome. AFP trajectory was monitored from preoperative to 6- and 12-month postoperative values, using time-varying Cox regression with AFP as a time-dependent covariate. The predictive accuracy of this time-updated model was compared with a static preoperative Cox model excluding postoperative AFP.

**Results:**

Among 1911 patients, AFP trajectories differed between recurrent and nonrecurrent cases. While preoperative AFP values were similar, recurrent cases exhibited higher AFP at 6 and 12 months. Multivariable analysis identified TBS (hazard ratio (HR):1.043 [95% confidence interval (CI): 1.002–1.086]; *p* = 0.039) and postoperative log AFP dynamics (HR:1.216 [CI 1.132–1.305]; *p* < 0.001) as predictors. Contour plots depicted TBS’s influence decreasing over time, while postoperative AFP became more predictive. The time-varying Cox model was created to update RFS predictions continuously on the basis of the latest AFP values. The preoperative Cox model, developed with age, AFP, TBS, and albumin-bilirubin score, had a baseline C-index of 0.61 [0.59–0.63]. At 6 months, the time-varying model’s C-index was 0.70 [0.67–0.73] versus 0.59 [0.56–0.61] for the static model; at 12 months, it was 0.70 [0.66–0.73] versus 0.56 [0.53–0.59]. The model was made available online (https://nm49jf-miho-akabane.shinyapps.io/AFPHCC/).

**Conclusions:**

Incorporating postoperative AFP dynamics into RFS prediction after HCC resection enhanced prediction accuracy over time, as TBS’s influence decreased. This adaptive, time-varying model provides refined RFS predictions throughout follow-up.

**Supplementary Information:**

The online version contains supplementary material available at 10.1245/s10434-025-17303-y.

Hepatocellular carcinoma (HCC) is a leading cause of cancer-related mortality globally.^1^ Hepatectomy remains the primary curative treatment option;^2^ however, long-term survival post-surgery is suboptimal, with 40–50% of patients experiencing early recurrence within 2 years.^3,4^ Accurate prognostic tools to predict HCC recurrence are essential to guide treatment decisions and optimize postoperative surveillance. Existing prediction models predominantly rely on preoperative factors, such as alpha-fetoprotein (AFP) levels and the tumor burden score (TBS)—a continuous variable calculated from tumor size and number—to estimate recurrence-free survival (RFS) post-hepatectomy.^5,6^

Serum AFP has been used as a biomarker for HCC since the 1970s^7^ and is routinely monitored postoperatively during outpatient follow-up to assess recurrence risk.^7,8^ Although other tumor markers, such as protein-induced by vitamin K absence or antagonist-II have also been associated with HCC,^7^ AFP remains the most commonly used marker related to HCC tumorigenesis.^9^ Elevated AFP levels often prompt further imaging to investigate recurrence,^8,10^ and while standardized guidelines from the National Comprehensive Cancer Network recommend post-resection imaging every 3–6 months for 2 years and every 6 months thereafter, along with AFP monitoring, there remains variability in how strictly these guidelines are followed and how clinicians interpret AFP dynamics to tailor individualized risk assessment. No model currently exists to predict recurrence on the basis of time-updated postoperative AFP values. “Time-updated” indicates prognostic models that recalculate or “update” at each relevant postoperative time point (e.g., at 6 months, 12 months) to incorporate the most recent AFP measurements. Since AFP, a protein expressed in HCC tumor cells, may be released by circulating or newly proliferating tumor cells after resection, relying solely on preoperative AFP levels limits prediction accuracy.^11^ As recurrence risk fluctuates over time, a time-updated model incorporating postoperative AFP levels may enhance prediction of recurrent disease.

To date, most prediction models use single, static data points; even studies highlighting postoperative AFP levels generally consider only a single time point, failing to track AFP changes over time.^12^ AFP alone may be insufficient to predict recurrence accurately; in turn, adding other tumor-related factors may improve risk assessment. Integrating TBS may offer a more comprehensive view of tumor burden, helping identify individuals who may benefit from additional imaging.^5^ We hypothesized that combining tumor burden with dynamic, time-updated AFP measurements throughout the postoperative period may provide a superior model that continually adapts to each patient’s evolving risk profile. Utilizing an international, multi-institutional database, we developed a time-updated prediction model that integrated postoperative AFP trends with tumor burden, allowing for time-updated, dynamic risk assessments to improve recurrence prediction during outpatient follow-up.

## Patients and Methods

### Study Population and Exclusion Criteria

Patients who underwent curative-intent liver resection for HCC between 2000 and 2023 were identified from an international, multi-institutional database. Patients who underwent a palliative resection or who had incomplete follow-up data were excluded. The study was approved by the institutional review board of each participating institution.

### Variables of Interest, Definitions, and Outcomes

Data were collected on patient demographics, including age, sex, the American Society of Anesthesiologists (ASA) physical status classification, body mass index (BMI), and preoperative cirrhosis status. Tumor staging was based on the 8th edition of the American Joint Committee on Cancer (AJCC) Staging Manual.^13^ Laboratory data included preoperative aspartate aminotransferase (AST; IU/L), alanine aminotransferase (ALT; IU/L), platelet count (× 10^3^/µL), albumin–bilirubin (ALBI) score,^14,15^ international normalized ratio (INR), and serum AFP levels (ng/mL). In addition, postoperative AFP levels were collected at 6 and 12 months, to capture changes over time. Tumor characteristics, including maximum tumor diameter, number of lesions, and microvascular invasion were documented. Surgical variables included surgical approach (open or minimally invasive), the extent of hepatectomy (with major hepatectomy defined as resection of more than three liver segments according to Couinaud’s classification),^16^ and whether adjuvant chemotherapy was administered. The tumor burden score (TBS), which accounts for both the maximum tumor size and the number of tumors, was calculated using the formula TBS^2^ = (maximum tumor diameter)^2^ + (number of tumors)^2^.^5^^,^^17^

The primary outcome was RFS, defined as the time from the date of curative-intent surgery to the date of recurrence, death from any cause, or last follow-up, whichever came first.

### Statistical Analyses

Continuous variables were presented as medians with interquartile ranges (IQR), while categorical variables were expressed as frequencies and percentages. Categorical variables were compared using the chi-squared test or Fisher’s exact test, and continuous variables were assessed using the Mann–Whitney *U* test or Kruskal–Wallis test, as appropriate.

To account for AFP changes over time, each patient’s AFP trajectory was retrospectively recorded from the preoperative period through 6 months and 12 months postoperatively (with complete data in all patients); these retrospectively obtained AFP values were then analyzed using a time-varying Cox regression analysis. Because some patients had zero or near-zero AFP levels, a log transformation with a unit offset (log (AFP + 1)) was employed to appropriately include all values in the model. The model incorporated each patient’s longitudinal AFP dynamics as a time-dependent covariate (“postoperative log AFP dynamics”) rather than as separate variables for preoperative, 6-month, and 12-month AFP values. This approach captured the cumulative effect of AFP level changes on RFS, providing a more comprehensive view of AFP’s influence throughout the postoperative period. The hazard ratios (HR) of “postoperative log AFP dynamics,” therefore, reflected the overall variation in AFP levels during follow-up rather than AFP values at individual time points. The correlation between AFP at each time point (preoperative, 6 months, and 12 months) and TBS was evaluated using Spearman’s correlation coefficient. Contour plots were generated to visualize the HR for RFS on the basis of TBS and AFP values at each time point, allowing an assessment of how the influence of AFP and TBS on RFS changed over time.

In the time-varying Cox model, RFS was predicted at each time point by sequentially incorporating AFP data available up to that time. For instance, at the preoperative stage, predictions relied solely on preoperative data to estimate RFS. At the 6-month stage, predictions incorporated both preoperative data and 6-month AFP levels. Similarly, at the 12-month stage, predictions included preoperative data and 6-month AFP and 12-month AFP measurements. This time-updated approach was compared at each stage with a static preoperative Cox model that did not incorporate any postoperative AFP data. C-index values were calculated at each time point to assess and compare the predictive accuracy of both models over time. For sensitivity analyses, this evaluation was repeated after excluding patients who received adjuvant chemotherapy, thereby considering the potential impact of adjuvant therapy on recurrence. For all analyses, statistical significance was set at a *p*-value of < 0.05. All statistical analyses were conducted using R version 4.3.1.

## Results

### Study Population

Among 1911 patients who underwent curative-intent surgery for HCC, median age was 67 [interquartile range (IQR): 59, 73] years with 1485 (77.7%) patients being male. A total of 837 (43.8%) patients had an ASA class > 2 (Table 1). Median BMI was 25.2 [IQR: 22.6, 28.4] kg/m^2^, and 851 (44.5%) patients had preoperative cirrhosis. Median ALBI score was −3.60 [IQR: −3.86, −3.24], and median preoperative log AFP was 2.38 [IQR:1.25, 5.01]. Major hepatectomy was performed in 699 (36.6%) patients. Median tumor size, calculated as the maximum diameter, was 4.80 [3.00, 8.10] cm, with a median tumor number of 1 [1,1]. Median TBS was 5.10 [3.35, 8.56]. Microvascular invasion was observed in 481(25.2%) patients. Adjuvant chemotherapy and immunotherapy were administered to 33 (1.7%) and 25 (1.3%) patients, respectively.

**Table 1:** Baseline characteristics of the study cohort: clinical and demographic variables

Variable	Overall (*N* = 1911)
Age, median [IQR]	67.0 [59.0, 73.0]
Male, yes, *n* (%)	1485 (77.7%)
ASA class > 2, yes, *n* (%)	837 (43.8%)
BMI, median, kg/m^2^ [IQR]	25.2 [22.6, 28.4]
Cirrhosis, yes, *n* (%)	851 (44.5%)
Platelet, × 10^3^/µL, median [IQR]	184 [137, 242]
Albumin, g/L, median [IQR]	41.0 [38.0, 44.0]
Total bilirubin, mg/dL, median [IQR]	0.62 [0.49, 0.91]
ALBI score, median [IQR]	−3.60 [−3.86, −3.24]
AST, IU/L, median [IQR]	38.0 [25.0, 61.0]
ALT, IU/L, median [IQR]	36.0 [23.5, 63.0]
INR, median [IQR]	1.05 [1.00, 1.10]
Log AFP, median [IQR]	2.38 [1.25, 5.01]
Tumor number, median [IQR]	1.00 [1.00, 1.00]
Tumor size, cm, median [IQR]	4.80 [3.00, 8.10]
TBS, median [IQR]	5.10 [3.35, 8.56]
AJCC T category < T2, *n* (%)	1458 (76.3%)
Microvascular invasion, yes, *n* (%)	481 (25.2%)
Regional lymph node metastasis, yes, *n* (%)	514 (26.9%)
Minimally invasive surgery, yes, *n* (%)	524 (27.4%)
Major hepatectomy, yes, *n* (%)	699 (36.6%)
Adjuvant chemotherapy, yes, *n* (%)	33 (1.7%)
Adjuvant immunotherapy, yes, *n* (%)	25 (1.3%)

Continuous variables, median [IQR]; categorical variable, number (%)

*AFP α*-fetoprotein, *AJCC* American Joint Committee on Cancer, *ALBI* albumin–bilirubin, *ALT* alanine aminotransferase, *ASA* American Society of Anesthesiologists, *AST* aspartate aminotransferase, *BMI* body mass index, *INR* international normalized ratio, *IQR* interquartile range, *TBS* tumor burden score

**Fig. 1:**
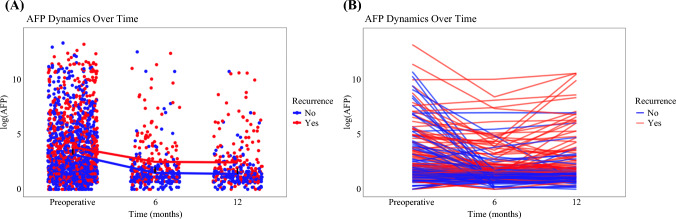
Dynamics of log AFP over time stratified by recurrence status; **A** mean trends in AFP over time stratified by recurrence status; the *x*-axis represents time points at preoperative, 6 months, and 12 months, and the *y*-axis represents log-transformed AFP values; blue dots and lines represent patients with no recurrence (“no”), while red dots and lines represent patients with recurrence (“yes”); the solid lines connect the mean log AFP values for each group (recurrence and no recurrence) at each time point, showing the overall trend in AFP dynamics for each group over time; **B** individual patient AFP trajectories over time; the *x*-axis represents time points at preoperative, 6 months, and 12 months, and the *y*-axis represents log-transformed AFP values; each line represents the AFP trajectory of an individual patient, with blue lines indicating no recurrence and red lines indicating recurrence; this panel focuses on the individual variation in AFP dynamics, allowing visualization of how AFP changes differ between patients over time

The dynamics of AFP over time, from preoperative to 6-month and 12-month postoperative measurements, are depicted in Fig. 1A, B. Mean trends in AFP were compared among patients who did and did not experience recurrence at each time point (preoperative, 6 months, and 12 months). While mean AFP levels were only slightly different preoperatively among patients with and without recurrence, postoperative measurements demonstrated consistently higher log AFP levels among patients who later developed recurrence (Fig. 1A). In addition, AFP trajectories over time were compared among recurrent and nonrecurrent cases within the same patient population (Fig. 1B). Although preoperative AFP values did not demonstrate notable differences, patients who experienced recurrence had higher AFP levels at both 6 and 12 months postoperatively (both *p* < 0.05).

### The Impact of Time-Dependent AFP Dynamics on RFS Prediction and the Relationship between AFP and TBS Over Time

**Fig. 2:**
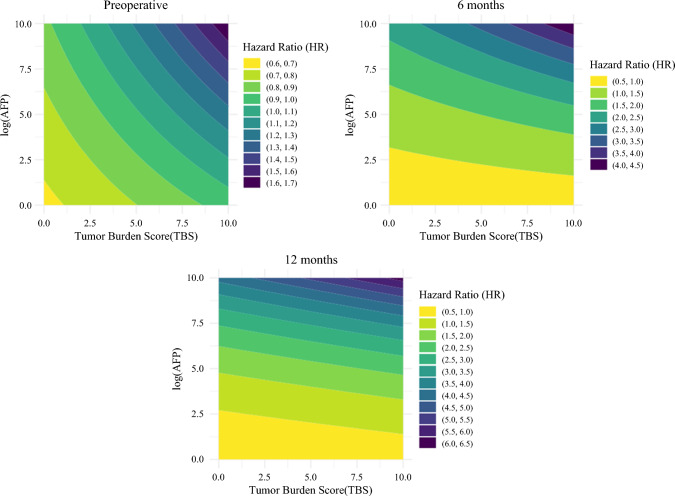
Contour plots showing the hazard ratios (HR) for tumor burden score (TBS) and log AFP at preoperative and postoperative time points (6 and 12 months); this figure consists of three plots that visualize the hazard ratios (HR) for recurrence-free survival (RFS) based on combinations of tumor burden score (TBS) and log-transformed AFP levels at preoperative, 6-month, and 12-month time points

The effect of postoperative AFP dynamics (“postoperative log AFP dynamics”) on RFS was evaluated using a Cox analysis with AFP as a time-dependent covariate (Table 2). On multivariable analysis, TBS (HR 1.043 [CI: 1.002–1.086]; *p* = 0.039) and postoperative log AFP dynamics (HR 1.216 [CI: 1.132–1.305]; *p* < 0.001) were strong predictors of recurrence. To further understand the interaction between these two factors, the correlation between TBS and log AFP from the preoperative to postoperative period was evaluated (Supplementary Fig. 1). Preoperative AFP and TBS had a Spearman correlation coefficient of 0.17 (*p* < 0.001), which decreased to 0.05 (*p* = 0.268) at 6 months and to 0.01 (*p* = 0.850) at 12 months, indicating a progressive weakening of the correlation over time. The impact of both TBS and AFP on the hazards of RFS was assessed at the preoperative period, as well as 6 months and 12 months postoperatively (Fig. 2). The contour plots demonstrated that both AFP and TBS had a strong influence on RFS preoperatively, but as time progressed, the influence of TBS diminished while the impact of postoperative AFP became increasingly more prominent. These findings underscored the importance of considering not only preoperative factors, such as TBS, but also postoperative factors, such as AFP dynamics, in assessing risk of recurrence in the short- and long-term postoperative period.

**Table 2:** Univariate and multivariable cox regression analysis incorporating time-dependent AFP dynamics for recurrence-free survival prediction (time-varying Cox model)

	Univariate		Multivariable	
Variable	HR (95% CI)	*P*-value	HR (95% CI)	*P*-value
Age	1.002 (0.990–1.015)	0.737	1.006 (0.992–1.020)	0.429
Male, yes	0.948 (0.678–1.325)	0.755	1.178 (0.776–1.789)	0.442
ASA class > 2, Yes	0.874 (0.663–1.151)	0.337	0.887 (0.634–1.241)	0.485
BMI, kg/m^2^	0.993 (0.962–1.025)	0.650	1.001 (0.965–1.039)	0.952
TBS	1.037 (1.006–1.068)	0.019	1.043 (1.002–1.086)	0.039
ALBI score	1.409 (1.059–1.876)	0.018	1.300 (0.926–1.061)	0.102
Preoperative log AFP	1.057 (1.006–1.111)	0.029	0.992 (0.926–1.061)	0.806
Postoperative log AFP Dynamic	1.209 (1.139–1.284)	< 0.001	1.216 (1.132–1.305)	< 0.001

*AFP α*-fetoprotein, *ALBI* albumin–bilirubin, *ASA* American Society of Anesthesiologists, *BMI* body mass index, *CI* confidence interval, *HR* hazard ratio, *TBS* tumor burden score

### Creation of a Time-Varying Cox Model Incorporating Time-Dependent AFP Dynamics

**Fig. 3:**
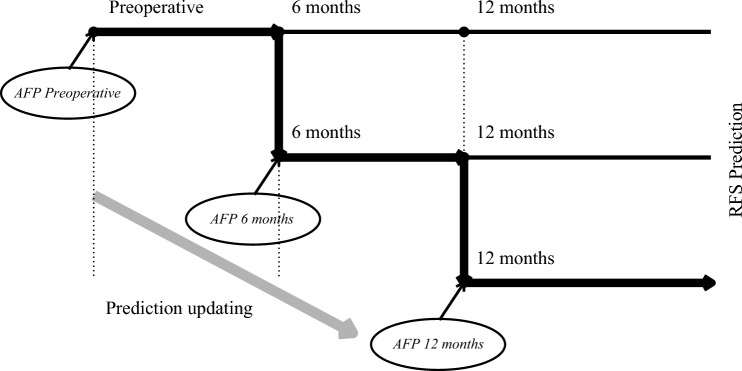
Concept of time-dependent AFP-based recurrence-free survival (RFS) prediction updating; this figure presents the concept of dynamic prediction updating for recurrence-free survival (RFS) based on alpha-fetoprotein (AFP) measurements at different postoperative intervals (preoperative, 6 months, and 12 months); each horizontal line represents a predictive trajectory based on AFP values at specific time points, with a key pathway illustrating the ongoing updating of the RFS prediction as new AFP data become available over time; the large arrow labeled “prediction updating” emphasizes the continuous and evolving nature of the model, which integrates longitudinal AFP changes to refine the RFS prediction

A real-time prediction model (“time-varying Cox model”) was developed to update RFS predictions at each postoperative period—immediately post-surgery, at 6 months, and at 12 months—on the basis of the AFP levels measured at each respective time point (Fig. 3). First, a baseline RFS prediction model (“preoperative Cox model”) was created using only preoperative factors in the multivariable Cox model (Supplementary Table 1). This analysis identified age (HR 1.009 [CI: 1.002–1.017]; *p* = 0.019), log AFP (HR 1.052 [CI: 1.021–1.084]; *p* < 0.001), TBS (HR 1.049 [CI: 1.031–1.068]; *p* < 0.001), and ALBI score (HR 1.433 [CI: 1.207–1.701]; *p* < 0.001) as factors predictive of RFS. To enhance predictive accuracy of RFS over time, postoperative AFP trends (“time-dependent AFP dynamics”) were incorporated into a time-varying Cox model on the basis of five factors: age, preoperative log AFP, TBS, ALBI score, and time-dependent AFP dynamics. This model allowed RFS predictions to be updated dynamically as new AFP measurements became available postoperatively.

### Predictive Power of the Time-Varying Cox Model

**Fig. 4:**
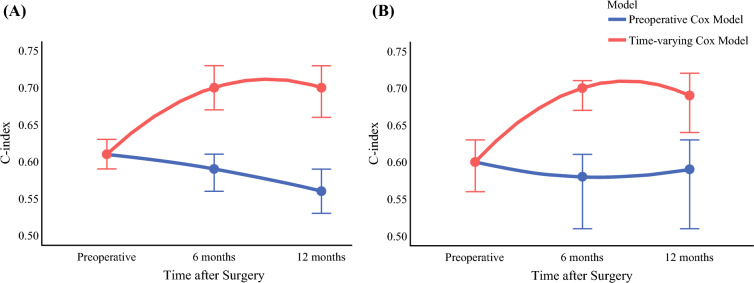
Comparison of preoperative and time-varying Cox model predictive accuracy for recurrence-free survival; this figure compares the predictive accuracy of preoperative and time-varying Cox models for recurrence-free survival (RFS) using C-index values at preoperative, 6-month, and 12-month postoperative time points; the blue curve represents the preoperative model, while the red curve represents the time-varying model; **A** displays the C-index values for the entire cohort; **B** shows a sensitivity analysis excluding patients who received adjuvant therapy; the preoperative model trends remain similar, while the time-varying model still demonstrates higher predictive accuracy over time

The predictive accuracy of the preoperative Cox model and the time-varying Cox model for RFS was compared using the C-index (Fig. 4A, B). The preoperative Cox model achieved a C-index of 0.61 [0.59–0.63] at baseline; however, at the 6-month mark, the time-varying Cox model demonstrated improved accuracy with a C-index of 0.70 [0.67–0.73] versus only 0.59 [0.56–0.61] for the static model based solely on preoperative factors. At 12 months, the time-varying Cox model maintained a C-index of 0.70 [0.66–0.73] versus 0.56 [0.53–0.59] for the preoperative Cox model (Fig. 4A). To account for the potential influence of adjuvant therapy on AFP levels, a sensitivity analysis was conducted that excluded all patients who received adjuvant therapy from the cohort (Fig. 4B). This analysis demonstrated similar trends: at baseline, the C-index was 0.60 [0.56–0.63] for the static preoperative model yet improved to 0.70 [0.67–0.71] at 6 months for the time-varying Cox model (versus 0.58 [0.51–0.61] for the preoperative Cox model) and to 0.69 [0.64–0.72] at 12 months (versus 0.59 [0.51–0.63]).

To facilitate clinical use, the time-varying Cox model was implemented as an online calculator (https://nm49jf-miho-akabane.shinyapps.io/AFPHCC/). Using this calculator, entering only preoperative data provided a baseline RFS prediction, while incorporation of AFP data at 6- and 12-month time points updated the RFS prediction at those time points.

## Discussion

Recurrence frequently occurs in HCC following curative-intent resection.^3^^,^^4^ To improve long-term outcomes, early identification of recurrence through tailored postoperative surveillance is essential. Adjusting surveillance intensity, including imaging evaluations, on the basis of individualized recurrence risk can optimize outcomes. While various prediction models have been reported, most rely solely on preoperative factors,^5^^,^^6^ and no real-time models have yet been developed that can update predictions as postoperative tumor-related factors evolve. AFP, a routinely measured tumor marker,^7^ helps assess recurrence risk during follow-up. However, postoperative AFP trends often do not correlate with preoperative AFP levels, potentially obscuring the true recurrence risk fluctuations if only preoperative AFP is considered. We hypothesized that incorporating postoperative AFP values into a time-updated model could offer a more accurate reflection of changing recurrence risk. The current study was, therefore, important as it addressed this gap by developing a time-varying Cox model that can periodically update RFS predictions, initially on the basis of preoperative factors such as AFP and TBS, with postoperative AFP values. This approach demonstrated superior predictive power over models based solely on preoperative factors. The analysis revealed that the influence of TBS on RFS decreased over time post-surgery, while postoperative AFP became increasingly significant. Notably, a time-varying model-based online calculator was developed, allowing real-time updates of RFS predictions, helping clinicians refine risk assessments on the basis of routine postoperative AFP levels.

Previous studies on RFS prediction for HCC after curative-intent resection have identified various predictors;^18^^,^^19^ however, these models generally rely on static factors or binary categories for post-treatment markers (e.g., high or low AFP), lacking a mechanism to continually update risk assessments with ongoing changes.^20^^,^^21^ Since recurrence risk can fluctuate during follow-up, continuously updating risk predictions to accurately reflect these changes is essential, especially given the high costs associated with imaging modalities such as contrast-enhanced computed tomography and magnetic resonance imaging.^21^^,^^22^ Addressing this need would enable more targeted and cost-effective surveillance. AFP, a well-known tumor marker^23^ generally absent in adult liver cells, is reactivated during oncogenesis, promoting cancer progression via multiple signaling pathways.^24^ AFP re-expression occurs mainly through hepatocyte proliferation, transcription factor activation under oxidative stress, and proto-oncogene activation, which can drive hepatocarcinogenesis.^25^^,^^26^ Following resection, AFP levels typically drop with tumor removal, yet AFP mRNA often persists in circulating tumor cells.^11^ Serum AFP levels are significantly higher in AFP mRNA-positive groups than in mRNA-negative groups, and elevated AFP postoperatively may signal the growth of residual or emerging tumor cells.^11^ Static preoperative models cannot capture these dynamic AFP changes. While postoperative AFP trends offer insights into recurrence risk, these data do not fully reflect the patient’s background or initial tumor characteristics. Ideally, a model that integrates both the baseline patient information and real-time AFP levels at each postoperative stage offers a more comprehensive approach. Using a time-varying Cox model, each patient’s recurrence risk can be tracked in real-time, and this methodology can be used to support dynamic, individualized predictions.

The current study demonstrated that, in time-varying Cox analysis, postoperative AFP dynamics serve as an independent risk factor for RFS. While TBS was a significant predictor preoperatively, its association with RFS weakened over time post-surgery, whereas AFP values at each postoperative stage became more influential. TBS, defined as the distance from the origin on a Cartesian plane with tumor size on the *x*-axis and the number of lesions on the *y*-axis,^17^ has been validated as a strong prognostic tool to stratify patients with primary and secondary liver tumors.^5^^,^^27^^,^^28^ However, following resection, recurrence risk depends more on the growth of residual tumor cells. From this perspective, the predictive power of the original tumor size and number decreases over time. Research has demonstrated that the tumor microenvironment is critical in HCC progression, with AFP driving carcinogenesis through mechanisms such as proliferation, angiogenesis, and invasion.^25^ AFP also has immunosuppressive effects that allow early malignant cells to evade immune surveillance, promoting cancer cell growth by activating survival pathways and inhibiting apoptosis, preventing cell death.^29^^–^^31^ Incorporating postoperative AFP into a predictive model offers a more detailed view of recurrence risk over time than static preoperative factors. The current study underscores the importance of AFP in real-time risk assessment and strongly suggests that a time-varying model incorporating postoperative AFP may enhance predictive accuracy to predict recurrence.

The current study was novel in that we used a time-varying Cox model, which addressed the limitations of traditional Cox models and demonstrated markedly higher predictive power, as evidenced by the high C-index. Traditional Cox models fail to account for changes in risk over time.^32^ Even if independent data points from the same patient—such as preoperative, 6-month, and 12-month postoperative measurements—were incorporated simultaneously into a single traditional Cox model, the model would still only provide a static snapshot of risk for each time point. Traditional Cox models treat each time point as isolated data inputs, disregarding any correlations or continuity between the values. Consequently, even if a correlation existed between preoperative data and 6-month postoperative data, the traditional Cox model would not account for this temporal continuity.^33^ In contrast, the time-varying Cox model can handle the evolution of a patient’s data over time within a single framework,^33^ allowing for the continuous tracking of risk throughout follow-up. This statistical approach allowed for higher predictive accuracy by capturing each patient’s evolving risk trajectory. Importantly, the model was made publicly available as an online calculator, allowing clinicians to input data at each time point (preoperative and 6 months and 12 months postoperative) to predict RFS from that point forward, enabling quick risk updates directly in the clinical setting.

Several limitations should be considered when interpreting the results of the current study. Differences in surgical protocols, techniques, treatments, and follow-up across institutions and countries may have introduced bias. In addition, only 12 patients (0.6%) received neoadjuvant therapy, limiting the ability to evaluate the influence on preoperative AFP levels. We also acknowledge that a formal internal or external validation of the time-varying model was not performed in the current study; prospective validation in an independent cohort is needed to confirm predictive performance. Although we examined only AFP, other markers, such as the ALBI score, may have improved the model’s accuracy. Incorporating other HCC-related tumor markers may be an area for future study. Updating preoperative models with real-time postoperative factors, such as AFP, to better predict recurrence risk allows clinicians to move beyond experience-based decisions toward a more systematic, data-driven framework for prognosis, patient management, and surveillance intensity.

In conclusion, prediction of RFS after curative-intent resection for HCC was markedly influenced by changes in AFP over time following surgical resection, while the impact of TBS on RFS gradually diminished. As time following surgery progressed, AFP values became more important, making AFP dynamics a critical predictive factor relative to RFS. By refining the traditional RFS prediction model to a time-varying Cox model that incorporated postoperative AFP dynamics, a real-time model capable of updating risk scores as time progresses was developed. This user-friendly model was made publicly available as an online calculator, allowing clinicians to make real-time, data-driven RFS predictions.

## Supplementary Information

Below is the link to the electronic supplementary material.

Supplementary file1 (DOCX 857 KB)
