# Supplementary material for: Enhancing Recurrence-Free Survival Prediction in Hepatocellular Carcinoma: A Time-Updated Model Incorporating Tumor Burden and AFP Dynamics
Source: Ann Surg Oncol. 2025 Apr 16;32(8):5648–56. doi: 10.1245/s10434-025-17303-y (PMC12222439; doi:10.1245/s10434-025-17303-y)
Supplement: Supplementary file 1 — Supplementary file1 (DOCX 857 KB) [file 10434_2025_17303_MOESM1_ESM.docx]

**Supplementary Figure 1. Correlation Between Tumor Burden Score (TBS) and AFP Levels at Preoperative and Postoperative Time Points (6 and 12 Months)**

This figure illustrates the relationship between Tumor Burden Score (TBS) and log-transformed AFP levels at three time points: preoperatively, 6 months postoperatively, and 12 months postoperatively. The blue line represents AFP levels at the preoperative time point, the orange line shows AFP levels at 6 months postoperatively, and the green line depicts AFP levels at 12 months postoperatively. Shaded areas around each line indicate the 95% confidence intervals. Spearman correlation coefficients and corresponding p-values are provided to assess the strength and significance of the correlation at each time point.

**Supplementary Figure 2. Example Use of the Online Real-time Time-varying RFS Prediction Calculator for HCC Patients Based on AFP Levels**

This figure demonstrates the use of an online calculator for predicting recurrence-free survival (RFS) in hepatocellular carcinoma (HCC) patients, based on AFP levels and other clinical data.

**Supplementary Table 1: Predictors of Recurrence-Free Survival in Cox Proportional Hazard Model: Univariate and Multivariate Analysis**

|  | **Univariate** | | **Multivariable** | |
| --- | --- | --- | --- | --- |
| **Variable** | **HR (95% CI)** | **p-value** | **HR (95% CI)** | **p-value** |
| Age | 1.005 (0.999 – 1.011) | 0.134 | 1.009 (1.002 – 1.017) | 0.019 |
| Male, Yes | 1.058 (0.896 – 1.249) | 0.508 | 1.194 (0.960 – 1.485) | 0.111 |
| ASA class>2, Yes | 1.059 (0.919 – 1.221) | 0.428 | 1.130 (0.940 – 1.359) | 0.194 |
| BMI, kg/m^2^ | 0.986 (0.970 – 1.002) | 0.095 | 0.993 (0.973 – 1.013) | 0.498 |
| Log AFP | 1.074 (1.049 – 1.100) | <0.001 | 1.052 (1.021 – 1.084) | <0.001 |
| TBS | 1.053 (1.039 – 1.068) | <0.001 | 1.049 (1.031 – 1.068) | <0.001 |
| ALBI score | 1.057 (1.006 – 1.111) | 0.029 | 1.433 (1.207 – 1.701) | <0.001 |

*Abbreviations.* AFP: *α*-Fetoprotein, ALBI: Albumin-Bilirubin, ASA: American Society of Anesthesiologists, BMI: Body Mass Index, CI: Confidence interval, HR: Hazard Ratio, TBS: Tumor Burden Score.
